# Supplementary material for: Association between oral microbiome and seven types of cancers in East Asian population: a two-sample Mendelian randomization analysis
Source: Front Mol Biosci. 2023 Nov 21;10:1327893. doi: 10.3389/fmolb.2023.1327893 (PMC10702768; doi:10.3389/fmolb.2023.1327893)
Supplement: Supplementary file 1 [file DataSheet1.ZIP › Supplementary Materials/MR plots of saliva/saliva═╝/Breast cancer/pheno.895_to_breast cancer_forest.pdf]

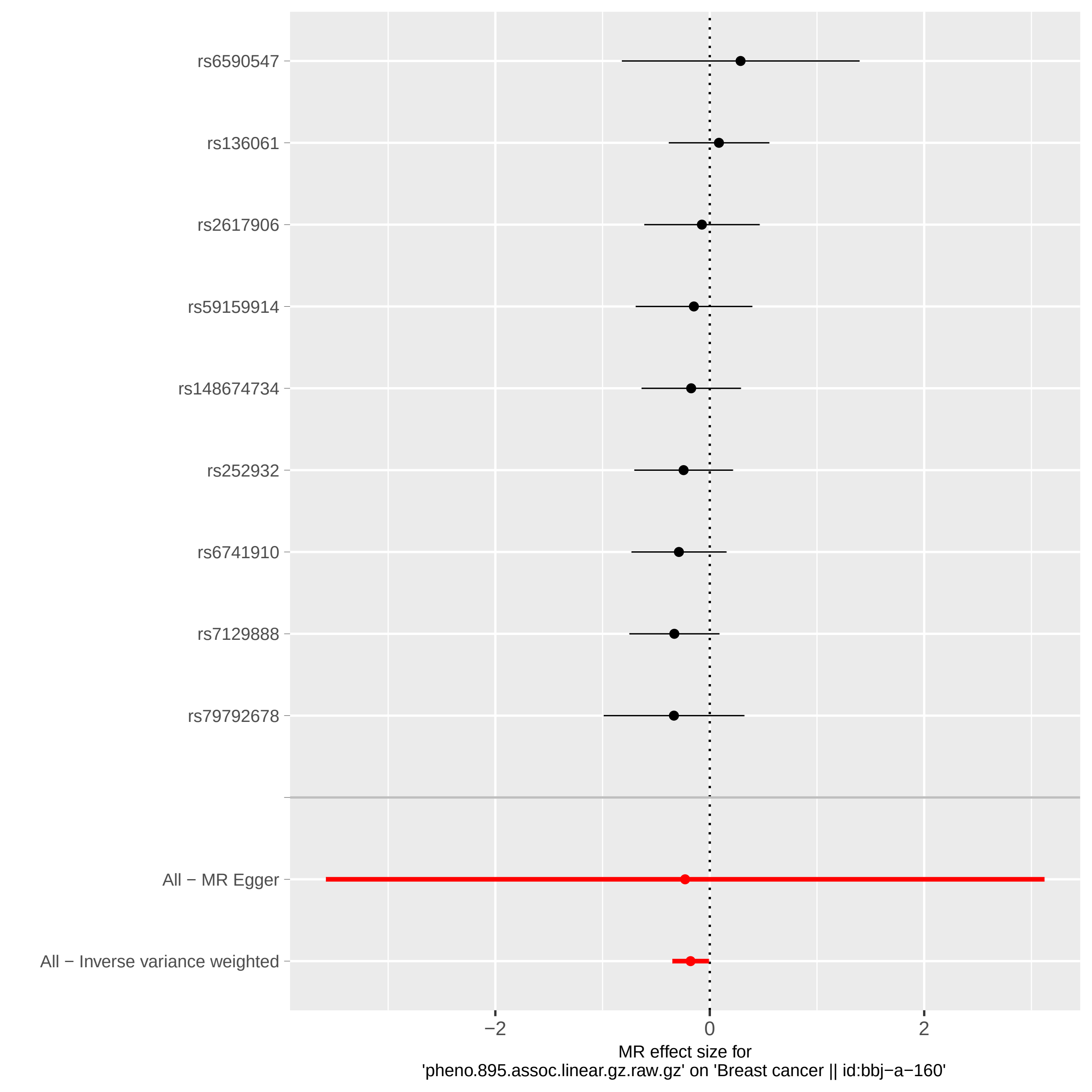

rs6590547
rs136061
rs2617906
rs59159914
rs148674734
rs252932
rs6741910
rs7129888
rs79792678
All − MR Egger
All − Inverse variance weighted
−2
0
2
MR effect size for
'pheno.895.assoc.linear.gz.raw.gz' on 'Breast cancer || id:bbj−a−160'
